# Influence of CYP2C9 and VKORC1 on Patient Response to Warfarin: A Systematic Review and Meta-Analysis

**DOI:** 10.1371/journal.pone.0044064

**Published:** 2012-08-29

**Authors:** Andrea L. Jorgensen, Richard J. FitzGerald, James Oyee, Munir Pirmohamed, Paula R. Williamson

**Affiliations:** 1 Department of Biostatistics, Shelley’s Cottage, University of Liverpool, Liverpool, United Kingdom; 2 Department of Molecular and Clinical Pharmacology, Block A: Waterhouse Building, University of Liverpool, Liverpool, United Kingdom; Tor Vergata University of Rome, Italy

## Abstract

**Background:**

Warfarin is a highly effective anticoagulant however its effectiveness relies on maintaining INR in therapeutic range. Finding the correct dose is difficult due to large inter-individual variability. Two genes, CYP2C9 and VKORC1, have been associated with this variability, leading to genotype-guided dosing tables in warfarin labeling. Nonetheless, it remains unclear how genotypic information should be used in practice. Navigating the literature to determine how genotype will influence warfarin response in a particular patient is difficult, due to significant variation in patient ethnicity, outcomes investigated, study design, and methodological rigor. Our systematic review was conducted to enable fair and accurate interpretation of which variants affect which outcomes, in which patients, and to what extent.

**Methodology/Principal Findings:**

A comprehensive search strategy was applied and 117 studies included. Primary outcomes were stable dose, time to stable dose and bleeding events. Methodological quality was assessed using criteria of Jorgensen and Williamson and data synthesized in meta-analyses using advanced methods. Pooled effect estimates were significant in most ethnic groups for CYP2C9*3 and stable dose (mutant types requiring between 1.1(0.7–1.5) and 2.3 (1.6–3.0)mg/day). Effect estimates were also significant for VKORC1 and stable dose for most ethnicities, although direction differed between asians and non-asians (mutant types requiring between 0.8(0.4–1.3) and 1.5(1.1–1.8)mg/day more in asians and between 1.5(0.7–2.2) and 3.1(2.7–3.6)mg/day less in non-asians). Several studies were excluded due to inadequate data reporting. Assessing study quality highlighted significant variability in methodological rigor. Notably, there was significant evidence of selective reporting, of outcomes and analysis approaches.

**Conclusions/Significance:**

Genetic associations with warfarin response vary between ethnicities. In order to achieve unbiased estimates in different populations, a high level of methodological rigor must be maintained and studies should report sufficient data to enable inclusion in meta-analyses. We propose minimum reporting requirements, suggest methodological guidelines and provide recommendations for reducing the risk of selective reporting.

## Introduction

Warfarin is a highly effective [1]–[5] and commonly used anticoagulant. However its effectiveness relies on attaining and maintaining a patient’s International Normalised Ratio (INR), a measure of clotting capability, within a therapeutic range. Predicting the dose necessary to achieve this, the so called ‘stable maintenance dose’, is difficult due to the drug’s narrow therapeutic index [6] and the large inter-individual variability in maintenance dose requirements [7].

Many clinical and environmental factors contributing to this variability have been identified, including age, body size, vitamin K intake, co-morbidities and co-medications. The focus of research over the last decade has shifted towards identifying genetic determinants of dose requirements, with several pharmacogenetic studies of warfarin response published annually. Many of these studies have identified significant associations with two genes in particular, the cytochrome-P450 gene CYP2C9 and the vitamin K epoxide reductase complex subunit 1 gene, VKORC1. Indeed, the evidence base for these associations is such that the FDA announced a change to warfarin labeling in 2007,and introduced dosing tables in 2010, to improve the dosing and hence the possible benefit-risk ratio of the drug [8].

Nonetheless, although it is widely accepted that genotype at CYP2C9 and VKORC1 affect dose requirements, it remains unclear exactly how genotypic information should be used when prescribing warfarin in practice. Indeed, the 2008 American College of Medical Genetics policy statement confirmed that there was insufficient evidence to recommend routine genotyping in warfarin-naive patients [9]. Further, the 2008 American College of Chest Physicians guidelines recommended against pharmacogenetic-based dosing until randomised data indicated that it is beneficial [10].

To determine from the literature how genotype will influence warfarin response in a particular patient is not an easy task because of the significant variation between studies in terms of the population studied, patient ethnicity, outcomes investigated and definition of those outcomes. It is perhaps fair to state that there is significant heterogeneity in study design and analysis approaches, and it cannot be assumed that all the studies are methodologically robust [11].

In order to methodically set out and assess the knowledge base accumulated so far, a systematic review of studies investigating association between variants in CYP2C9 and VKORC1 and warfarin response was undertaken. To comprehensively assess the reliability of each piece of evidence, the checklist of methodological quality for pharmacogenetic studies developed by Jorgensen and Williamson was applied [11]. Subject to the results of this assessment, data were synthesized by way of meta-analysis applying the specialist methods of Minelli [12] and Salanti [13], which represent some of the most advanced methods developed for synthesising evidence from genetic association studies, to ensure the most efficient use of available data, thus maximizing power.

At the time of planning our review, we identified that a systematic review of the role of CYP2C9 variants on clinical outcomes in warfarin-treated patients had been undertaken previously in 2003 [14]. However, a large number of warfarin pharmacogenetic studies had been published annually since 2003, meaning that our review included several more studies investigating CYP2C9 variants as well as those investigating the role of VKORC1 variants, or both. More recently, an ‘Analytic validity, Clinical validity, Clinical utility, and Ethical, legal, and social implications’ (‘ACCE’) review of allele testing to inform warfarin dosing included meta-analyses estimating the effect of variants in both CYP2C9 and VKORC1 on response to warfarin [15]. However, although the review is informative and addresses several key clinical questions, the report does not describe the search strategy employed in identifying included studies and the heterogeneity in effect estimates between studies was not evaluated or investigated. In our systematic review, a structured search strategy was adopted to ensure that all relevant studies were identified and that any meta-analyses conducted reflect up to date information from all available sources.

Variants in another gene, CYP4F2, have also been associated with warfarin dose requirements in several studies. However, the evidence base for this gene was very small at the time of planning our review and consequently we chose to focus our review only on CYP2C9 and VKORC1. Subsequently, a systematic review and meta-analysis has been published by another research group on the influence of CYP4F2 on warfarin dose requirements [16].

It was anticipated that formally reviewing all available evidence on each SNP-outcome association, including a rigorous assessment of methodological quality, study design and characteristics, and setting out the findings in an orderly manner would enable a fair and accurate interpretation of which variants affect which outcomes, in which patients, and to what extent. We also hoped to highlight any gaps requiring further research.

## Results

### Identification of Included Papers

**Figure 1:**
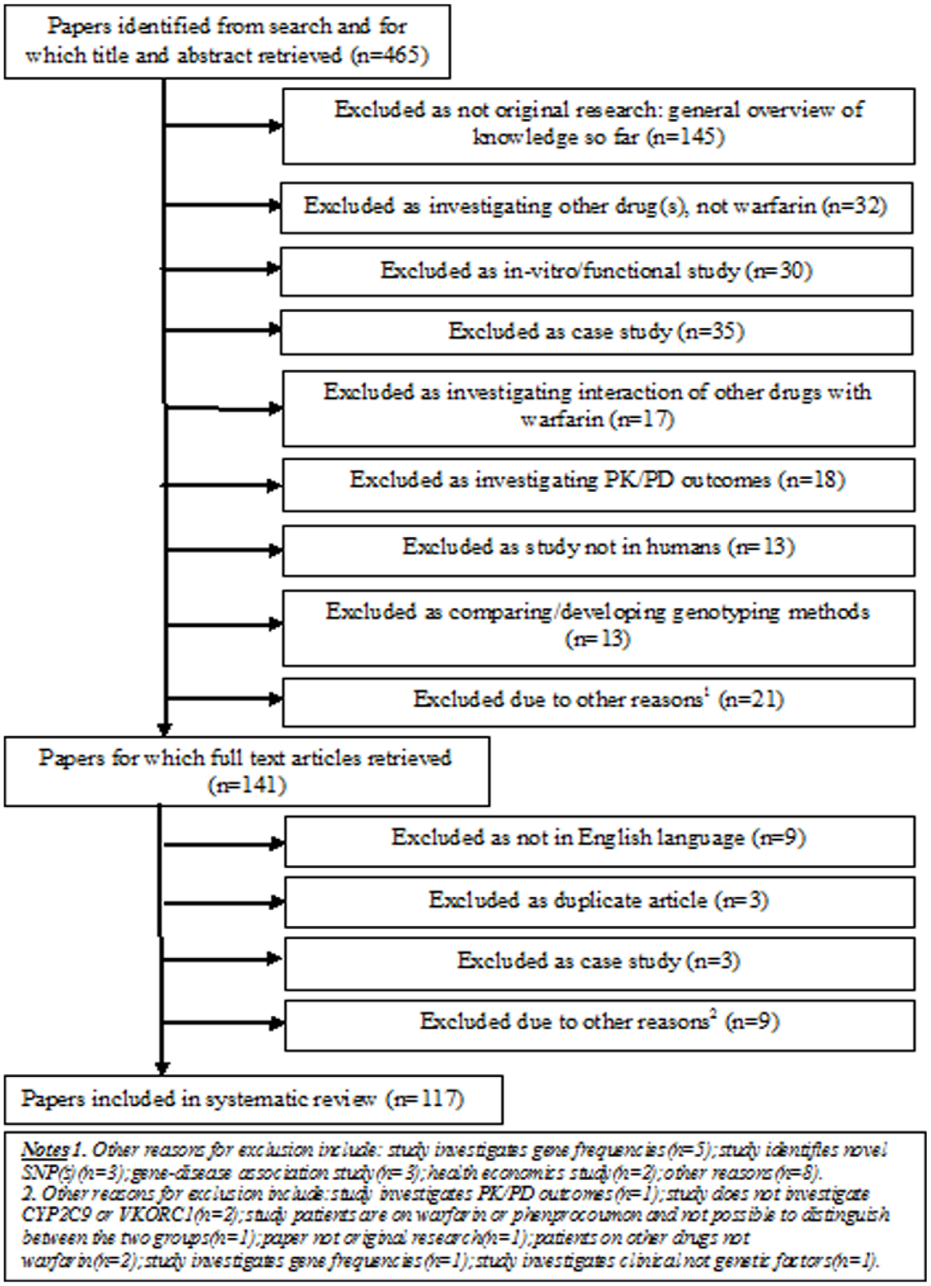
QUORUM flowchart.

The search strategy is summarized in Table 1. A Quorum flowchart is given in Figure 1. 117 studies were included in the systematic review - the full list, together with study characteristics and references, is available on request.

**Table 1:** Search Strategy.

Number	Search Term
1	warfarin.mp.
2	s-warfarin.mp.
3	r-warfarin.mp.
4	gene$.mp.
5	geno$.mp.
6	Haplotyp$.mp.
7	variant.mp.
8	allel$.mp.
9	SNP$.mp.
10	polymorphism$.mp.
11	CYP2C9.mp.
12	CYP2C9$.mp.
13	VKORC1.mp.
14	vitamin K epoxide reductase complex subunit.mp.
15	cytochrome p450.mp.
16	cytochrome p-450.mp.
17	cytochrome-p450.mp.
18	1 or 2 or 3
19	4 or 5 or 6 or 7 or 8 or 9 or 10
20	11 or 12 or 13 or 14 or 15 or 16 or 17
21	18 and 19 and 20

Notes.

1. mp = title, original title, abstract, name of substance word, subject heading word.

2. $ = any ending to the word.

### Associations Investigated by Included Studies

Details of which variants were investigated for association with each of the primary and secondary outcomes, and in which studies, are given in Table 2 and Table 3. Several studies did not investigate any of the primary or secondary outcomes.

**Table 2:** Studies investigating association between CYP2C9 variants and each outcome.

Outcome	Variant	Number ofstudiesinvestigatingassociation	Study(ies) investigated
Stablemaintenancedose	*2	55^1^	[17],[23],[42],[109],[137],[101],[95],[27],[107],[73],[93],[66],[43],[114],[138],[31],[75],[21],[108],[32],[37],[96],[28], [30], [39], [41], [44], [46], [48], [49], [51], [54], [58], [62], [63], [67], [68], [70], [81], [82], [85], [86], [89], [100], [103]-[105], [110], [112], [117], [120], [123], [124], [139], [140]
	*3	65^1^	[119],[23],[42],[109],[137],[101],[127],[113],[39],[100],[95],[27],[141],[112],[107],[93], [66],[89],[43],[19],[138],[75],[21], [103],[32],[37],[70],[96],[17], [25], [28], [31], [41], [44], [46], [48]-[51], [53], [54], [56], [58], [61]-[63], [65], [67], [68], [73], [81], [82], [85], [86], [104], [105], [108], [110], [117], [120], [123], [124], [128], [139], [140]
	*5	1	[31]
	*11	1	[38]
	Haplotypes	1	[102]
Time tostable dose	*2	9	[17], [32], [48], [49], [61], [63], [79], [82], [93]
	*3	9	[17], [32], [48], [49], [61], [63], [79], [82], [118]
	*13	1	[118]
	*14	1	[118]
BleedingEvents	*2	12	[32], [40], [43], [48], [49], [63], [79], [82], [87], [116], [117], [120]
	*3	16	[25], [32], [40], [43], [48], [49], [53], [61], [63], [79], [82], [87], [116], [117], [119], [120]
	*5	1	[82]
	*6	1	[82]
	*10	1	[82]
	*11	1	[82]
INR>4 duringfirst week	*2	2	[63], [95]
	*3	2	[63], [95]
Time totherapeuticINR	*2	7	[32], [40], [63], [79], [82], [95], [120]
	*3	7	[32], [40], [63], [79], [82], [95], [120]
Percentagetime intherapeuticrange	*2	4	[48], [49], [79], [96]
	*3	4	[48], [49], [79], [96]
Warfarinresistance	*2	1	[63]
	*3	1	[63]
Warfarinsensitivity	*2	1	[63]
	*3	1	[63]

Notes.

1. 10 of these studies investigated association between CYP2C9*2 and CYP2C9*3 combined genotype and stable dose.

**Table 3:** Studies investigating association between VKORC1 and each outcome.

Outcome	Variant	Number of studiesinvestigating association	Study(ies) investigated
**Stable maintenance dose**	rs9934438	15	[20], [36], [44], [52], [56], [61], [82], [86], [100], [105], [119], [125], [128], [137]
	rs9923231	17	[18], [24], [27], [50], [54], [58], [63], [77], [83], [88], [91], [128]
	rs7196161	1	[101]
	Asp37Tyr	1	[111]
	rs8050894	5	[51], [82], [83], [88], [105]
	rs7294	5	[63], [82], [105], [125], [128]
	47G>C	1	[127]
	113A>C	1	[127]
	1338A>G	1	[127]
	1442–1443 CCCGC insertion	1	[127]
	1413A>G	1	[113]
	136T>C	1	[113]
	124C>G	1	[113]
	837T>C	1	[113]
	343G>A	1	[113]
	rs2884737	1	[105]
	rs17708472	1	[105]
	rs2359612	5	[41], [63], [82], [83], [105]
	rs17886199	1	[83]
	rs17878338	1	[83]
	rs10871454	1	[46]
	Haplotypes	5	[26], [49], [72], [80], [97]
**Time to stable dose**	rs9934438	3	[61], [82], [118]
	rs7294	1	[63]
	rs2359612	1	[63]
	rs9923231	1	[63]
	Haplotypes	1	[49]
**Bleeding events**	rs9934438	2	[61], [82]
	rs7294	1	[63]
	rs2359612	1	[63]
	rs9923231	1	[63]
	Haplotypes	2	[49], [87]
**INR>4 during first week**	rs7294	1	[63]
	rs2359612	1	[63]
	rs9923231	1	[63]
**Time to therapeutic INR**	rs9934438	1	[82]
	rs7294	1	[63]
	rs2359612	1	[63]
	rs9923231	1	[63]
	Haplotypes	1	[87]
**Percentage time in therapeutic range**	Haplotypes	1	[49]
**Warfarin resistance**	rs7294	1	[63]
	rs2359612	1	[63]
	rs9923231	1	[63]
**Warfarin sensitivity**	rs7294	1	[63]
	rs2359612	1	[63]
	rs9923231	1	[63]

### Quality Assessment

Each of the criteria set out by Jorgensen and Williamson [11] were considered in turn, and the main findings are discussed below.

### Choosing Which Genes and SNPs to Genotype

All but one study provided a reason for choosing the gene(s) investigated, although for one [17] a reason was only provided for CYP2C9 and not VKORC1, within which SNPs were found associated with stable dose. For the study [18] where no reason was provided, the study report was in the form of a letter, hence necessarily brief. Nonetheless, the genes reported were also significantly associated with dose. Consequently, both these studies are deemed at risk of selective reporting. Two further studies [19], [20] are deemed at risk of selective reporting because they reported results for a subset of investigated genes only.

A further four studies[18], [21]–[23] were also deemed at risk, due to not providing sufficient justification for their choice of SNPs, which were all statistically significant, whilst another two [20], [24] are at risk due to reporting results for a subset of investigated SNPs only, all of which were statistically significant.

### Sample Size

The median sample size was 162 (IQR: 91–219), meaning that most studies were at risk of being underpowered [11]. None of the studies provided details of the a priori power for a range of allele frequency-effect size combinations, leaving the reader uninformed about the extent of power available and the likelihood of any non-significant results being false-negatives.

### Study Design

78 studies were retrospective cohorts, 34 were prospective, two were case-control studies and three were randomised controlled trials. For the two case-control studies, although the case and control groups were both clearly defined, there was no mention that the two groups were genotyped in mixed batches; clearly, separate genotyping could potentially bias the results.

### Reliability of Genotypes

The genotyping methods for some or all genes were not described in five papers [18], [25]–[28] making it difficult to assess the accuracy of genotyping. Only 37 studies [17], [24], [29]–[63] mentioned genotype quality control procedures, and therefore the genotyping results in the remaining 80 studies should potentially be interpreted with caution. Only 43 studies [17], [32], [35], [39], [44], [45], [47], [48], [51], [53], [54], [56]–[60], [62]–[88]compared genotype frequencies of all investigated SNPs to those previously published for the same population, a relatively simple way of highlighting problems with genotyping.

Of the 78 retrospective and two case-control studies, only six [31], [47], [49], [51], [89], [90] mentioned that genotyping personnel were blinded to outcome status. A further study [35] mentioned that those collecting clinical data were blinded to genotype status. Ideally, genotyping personnel should have been blinded to outcome status to minimise the risk of introducing bias during the genotype calling procedure.

### Missing Genotype Data

Missing genotype data were mentioned in 20 papers [22], [23], [30], [36], [41], [62], [63], [68], [70], [72], [82], [83], [86], [91]–[97]. Only one of these studies [70] described checking that missingness was at random, therefore the remaining 19 studies were at risk of non-random missing data, which could bias results. The 97 studies not describing any missing data were also at risk of this, since if there were missing data, then they may not have been missing at random.

### Population Stratification

No study mentioned undertaking tests for population stratification, and none adjusted for any potential cryptic population stratification, placing all at potential risk from confounding due to population stratification. Further, although patients formed more than one distinct ethnic group in 37 studies [17], [23], [29], [31], [35]–[37], [40], [45], [47], [53]–[55], [58], [63], [65], [66], [69], [71], [76], [78], [80], [81], [83], [84], [87], [89], [91], [98]–[105], only 21[17], [31], [36], [37], [47], [54], [58], [63], [65], [66], [76], [78], [80], [81], [83], [97]–[101], [104] stratified their analyses accordingly. The remaining 16 studies are at particular risk from confounding.

### Hardy-Weinberg Equilibrium (HWE)

Only 49 studies [17], [19], [26], [27], [30], [32], [35], [36], [39], [41], [42], [47], [48], [51], [54], [56], [58]–[63], [67]–[69], [72], [73], [79], [80], [82]–[87], [91], [95]–[97], [100], [102], [104]–[110] reported testing for HWE at all SNPs investigated, whilst a further two [24], [111] tested for HWE for a subset of SNPs only. However, tests for HWE were undertaken by us prior to conducting the meta-analyses.

### Mode of Inheritance

Twenty-eight studies made a specific assumption regarding the underlying mode of inheritance. Of these, only one [51] provided justification, whilst another [36] chose to assume a dominant mode on the basis that the number of mutant-types was small. For the remaining 26 studies [17], [23], [27], [31], [32], [37], [39], [40], [42], [44], [49], [50], [52], [63], [67], [69], [74], [77], [78], [84], [103], [104], [112]–[115] there is a risk of within-study selective reporting where several analyses under different modes of inheritance may have been conducted with only the most statistically significant being reported. The same is true for eight studies [22], [35], [70], [89], [95], [96], [116], [117] that compared various combinations of genotype groups with no apparent justification.

### Choice and Definition of Outcomes

There was large variation in definition of stable dose (Table 4). Of the 76 studies investigating this outcome, 21 did not provide a definition whilst for the remaining 55 studies, there were 34 different definitions.

**Table 4:** Definitions of stable dose in included papers.

Study	Definition of stable dose
[119]	Mean of doses for 3 consecutive clinic visits with INR in range
[23], [61]	Unchanged dose that gave therapeutic INR for 7 consecutive days (or 6 consecutive days where that didn't happen)
[128]	AC stably controlled with INR between 1.6 and 2.6
[48], [111]	INR in range at >/ = 4 consecutive clinic visits
[42]	Mean weekly dose required across 6 clinic visits after therapeutic INR already achieved
[20]	Unchanged dose for at least 6 months
[80], [106]	Same dose for >1 month with INR between 2–3
[101]	Mean of 2 recent doses over a period when 2 consecutive stable INR values were documented
[66], [125]	Dose required to achieve INR in therapeutic range for last 2 clinic visits at the same daily dose
[24], [39]	Stable INR (+/−10%) for at least 3 months on constant warfarin dose
[26], [27]	Constant dose taken at 3 consecutive clinic visits over a minimum period of 3 months, with INR within 2–3
[58]	Constant dose taken at more than 3 consecutive clinic visits over a minimum period of 3 months, with INR within 2–3
[77]	Constant dose for at least 3 weeks
[112]	Not specifically defined although patients were only recruited if they had been on maintenance therapy for >6 months with a stable INR within range during the last two clinic visits
[138]	Stable dose for at least 3 consecutive clinic visits prior to recruitment, and remained on that dose throughout the 4 week follow-up period
[31]	Average of last 2 doses taken. As mean difference between the 2 doses was relatively small, authors concluded they were all on stable dose
[108]	Stable dose with INR value varying no more than 15% at last 3 visits
[91]	A dose that did not vary by more than 10% between 3 consecutive clinic visits, over a minimum period of 8 weeks. INR had to be in range at those visits, although at one of those visits INR was allowed to be 0.2 above or below the target range.
[17], [30], [32], [38], [46], [51], [54], [62], [63], [86], [95], [102], [104], [114], [123]	3 consecutive clinic visits for which INR measurements were within therapeutic range for the same mean daily dose
[37]	<10% fluctuation in dose over preceding 4 wks prior to recruitment
[70]	Stable(+/−20%) INR values for at least 4 clinic visits on the same daily dose for at least 1 month before recruitment
[100]	On warfarin for at least 1 month and INR now in range
[65]	Constant warfarin dose at visits over a minimum period of 3 months, with INR in range (2–3)
[50]	Constant warfarin dose at visits over a minimum period of 3 months, with INR in range (1.5–3)
[56]	Constant warfarin dose for at least 3 consecutive clinic visits over a minimum period of 3 months, with INR in range (1.5–3)
[53]	Dose patients were on when their INR was between 2–4 at the 6 month follow-up time-point
[105], [117]	Dose required to achieve the patient's target INR
[21]	Dose needed for INR to be in range at 2 consecutive clinic visits (within minimum of 48 hrs interval) provided the dose was the same for the 5 days before first INR in range
[82]	Defined as the first dose that leads to a stable INR over three consecutive visits following initiation of the drug. These INR measurements encompassed a period of at least 2 weeks, with a maximum difference between the mean daily dosages of 10%
[25]	Mean dose required to achieve target INR range
[44]	The dose achieved on day 8 or later that was associated with ≥2 INRs within 15% of therapeutic range measured ≥1 week apart
[110]	Dose leading to therapeutic INR values between 2.5 and 3.5 for at least 3 months
[85]	Last three INR measurements considered stable by doctors, whether or not they correspond to the patient’s target INR.
[83]	Average dose after achieving therapeutic INR
[18], [19], [41], [43], [52], [67], [68], [72], [73], [75], [88], [89], [93], [96], [103], [109], [113], [120], [124], [127], [141]	not given

There was also variability in the definition of stable dose for the studies investigating time to achieving stability (Table 5). Five studies used the same definition [17], [32], [36], [63], [118] whilst the remaining four [48], [61], [79], [82] each used a different definition. The definition of a bleeding event also varied (Table 5). Of the fifteen studies investigating this outcome, one [119] provided no definition, whilst for the remaining fourteen studies there were nine different definitions. For the nine studies investigating the outcome of time to achieving therapeutic INR, one [120] did not provide a definition, whilst for the other eight studies three different definitions were used (Table 5). No definition was required for the outcome of INR>4 during the first week and time within therapeutic range, whilst only one study [63] investigated the outcomes of warfarin sensitivity or resistance.

**Table 5:** Definitions of time to stable dose, bleeding events and time to therapeutic INR.

Outcome	Studies	Definition
**Time to stable dose**	[61]	Unchanged dose that gave therapeutic INR for 7 consecutive days, or 6 consecutive days where that didn't happen
	[82]	Average dose after achieving therapeutic INR
	[48]	INR in range at >/ = 4 consecutive clinic visits
	[17], [32], [36], [63], [118]	3 consecutive clinic visits for which INR measurements were within therapeutic range for the same mean daily dose
	[79]	Two consecutive INR values, 7 days apart, in therapeutic range, without any intervening dose alteration
**Bleeding Events**	[32], [49]	Serious and life-threatening bleeds as defined in Fihn et al. [142]
	[53]	Three separate analyses undertaken: mild (bleeding not requiring additional testing, referral and outpatient visits); moderate (bleeding requiring medical evaluation/blood transfusion of 2 units or less); serious (bleeding requiring surgical or angiographic intervention, transfusion of 3 or more units of blood, or leading to irreversible sequale)
	[43]	Three separate analyses undertaken: minor bleeds (hematoma, microhematuria, mild epistaxis); moderate bleeds (hematoma, abundant epistaxis, hematuria) and severe complications (melena, macrohematuria)
	[25], [61], [79], [116], [120]	Any bleeding events during follow-up
	[117]	Serious bleeding requiring hospital care but excluding anyone having had thrombolysis, surgery or trauma immediately before bleed
	[40], [48]	Bleeding requiring re-hospitalisation or death
	[84]	Two separate analyses undertaken, one of minor bleeds (minor nosebleeds, microscopic hematuria, mild bruising, and mild hemorrhoidal bleeding) and one of major bleeds (serious, life-threatening and fatal bleeds as defined by Fihn et al. [142])
	[63]	All adverse events assessed for causality and events categorized as definitely, probably, possibly or unlikely to be related to warfarin. Haemorrhagic complications defined as major or minor according to classification provided by Fihn et al [142]. Only events considered to be possibly, probably or definitely associated with warfarin included in the analyses. Two separate analyses undertaken: one for all bleeding events and one for major bleeding events only.
	[87]	Major or minor bleeding event, according to criteria of the Second Copenhagen Atrial Fibrillation, Aspirin and Anti-coagulation study [143]
	[119]	No definition given
**Time to therapeutic INR**	[32], [48], [63], [73], [79], [87]	Time to first occurrence of an INR in therapeutic range
	[95]	Time to the first in a series of at least 3 consecutive therapeutic INRs on a stable dose
	[40]	Time to at least 2 consecutive INRs between 1.8 and 3.2 measured at least 7 days apart whilst on the same dose
	[120]	Not given
**Warfarin sensitivity**	[63]	A dose of ≤1.5 mg/day on three successive clinic visits.
**Warfarin resistance**	[63]	A dose of >10 mg/day on three successive clinic visits.

Only nineteen studies [35], [45], [47], [53], [56], [58], [59], [63], [79], [80], [82], [85]–[88], [120]–[123] explicitly justified their choice of outcomes; however for a further 30 studies [23], [26], [27], [29]–[31], [37], [39], [41], [42], [50]–[52], [60], [65], [67], [68], [75], [76], [78], [91], [98], [100], [101], [107], [111], [112], [117], [124], [125], although no explicit justification was provided, outcomes were in line with the main aim of the study as conveyed in the paper’s introduction. The remaining studies are at a particular risk of selective reporting of outcomes, although it is not possible to conclude for sure that any of the studies are completely free from this risk.

### Compliance with Treatment

Only four studies mentioned assessing compliance with treatment. Of the remaining studies, one [126] stated that ‘compliance was reasonably excluded’, but did not explain how whilst another [72] stated that a decision was made not to assess compliance, without justification. A further study [118] included history of noncompliance as an exclusion criterion. Of the four studies that assessed compliance, three [57], [74], [82] did not adjust their analyses for extent of compliance whilst this was not relevant in the fourth study [34] due to all patients reportedly being compliant.

### Meta-analyses

#### CYP2C9*2 and stable dose

**Figure 2:**
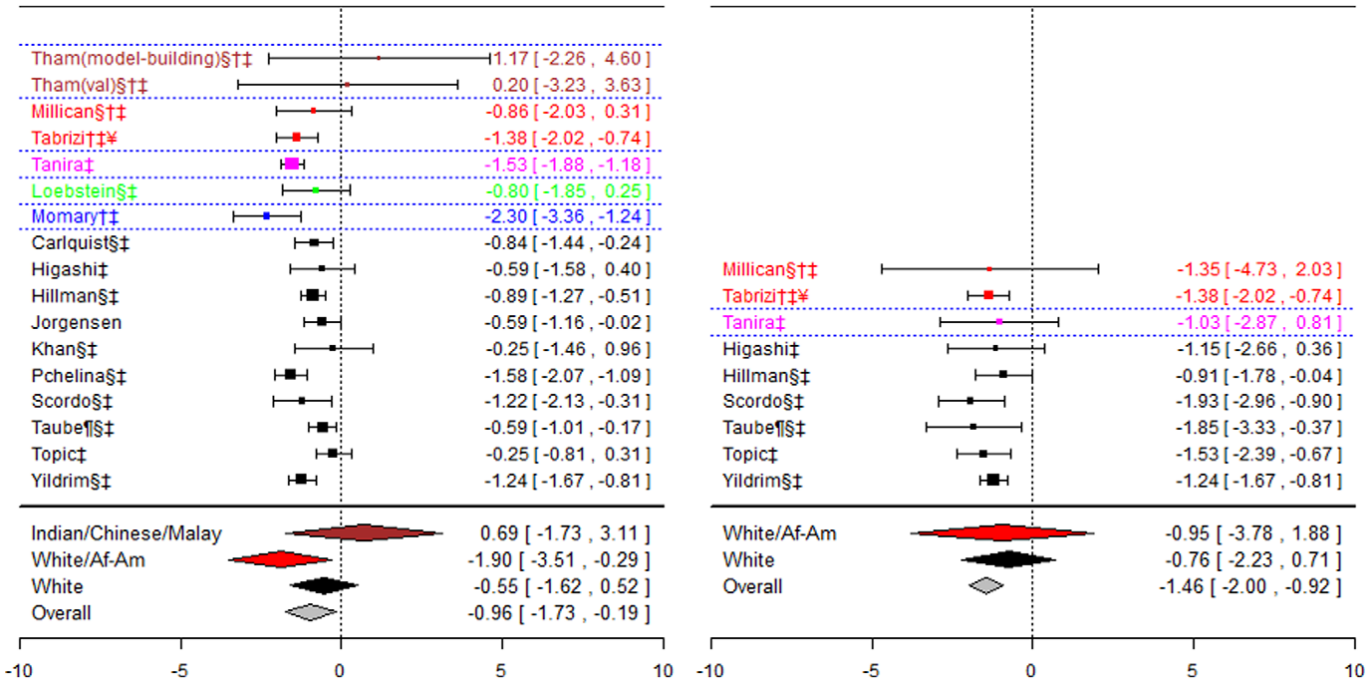
Forest plots for association between CYP2C9*2 and stable dose. Effect estimates are differences in means and 95% confidence intervals. ¶: Ethnicity of patients is unclear, although likely to be predominantly White so included in sensitivity analysis of White ethnic group. §: Paper does not mention genotype quality control procedures, so reliability uncertain.†: Paper does not mention tests for population stratification, which is of concern since more than one ethnic group included.‡: Paper does not mention assessing compliance with treatment. ¥: Studies reported results assuming a dominant mode of inheritance. The effect size estimated is therefore for heterozygotes and mutant-type homozygotes combined versus wild-type homozygotes.

45 studies investigated this association. 29 were excluded: four because data was only presented graphically [41], [62], [82], [124], 18 because insufficient data was presented [21], [27], [28], [31], [42], [44], [46], [47], [49], [58], [66], [75], [81], [85], [86], [105], [123], [124], although for one of these [46] the patients were from a study already included in the meta-analysis [32], and seven [17], [30], [93], [95], [104], [114], [120] since data was summarised as medians rather than means. Results from these twenty-nine studies are qualitatively consistent with the conclusions of the meta-analysis. Data for the remaining 16 studies are presented in Figure 2.

For the White and African-American ethnic origin group the pooled effect was significant for the first genotype contrast (−1.90(−3.51;−0.29) mg/day), but not for the second. The pooled effect estimate for the two cohorts including Indian, Chinese and Malay patients was not-significant, as were the two genotype contrasts for the White ethnic group. However, for the latter group there was significant heterogeneity between studies for the heterozygotes versus wild-types contrast (I^2^∶53%).

#### CYP2C9*3 and stable dose

**Figure 3:**
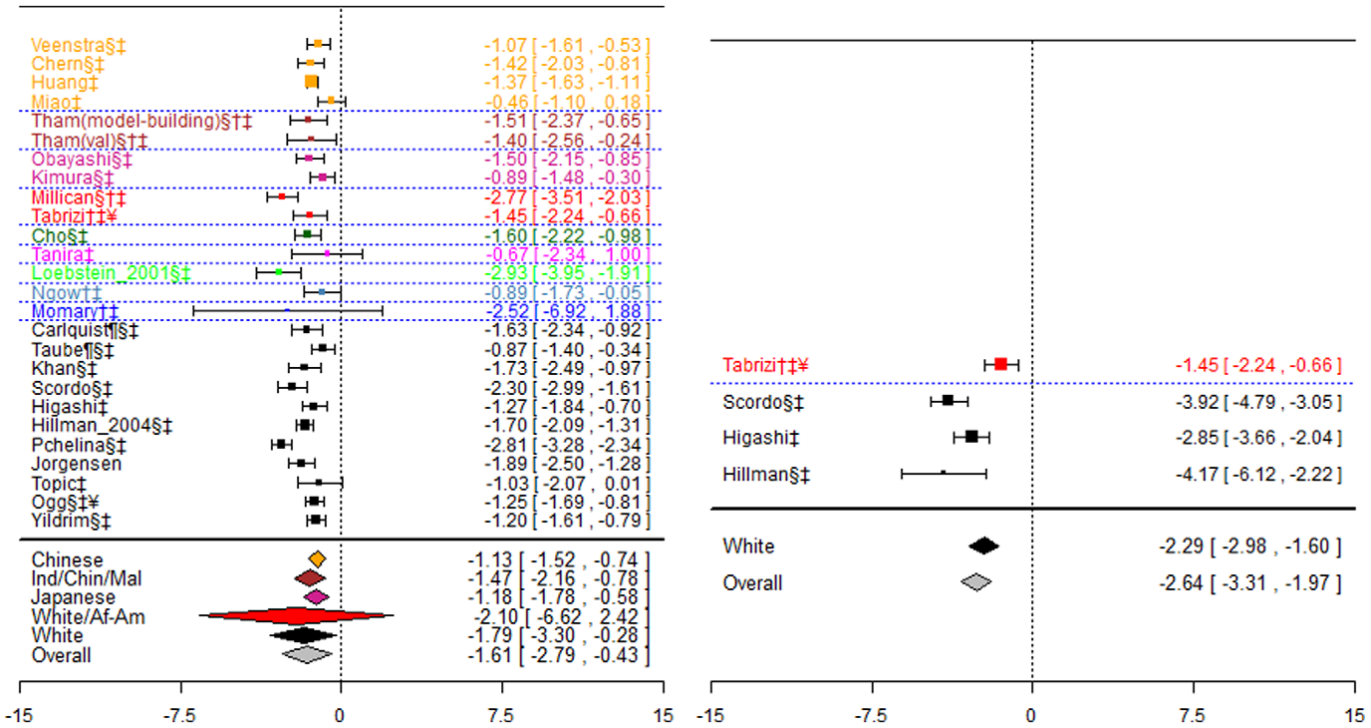
Forest plots for association between CYP2C9*3 and stable dose. Effect estimates are differences in means and 95% confidence intervals. ¶: Ethnicity of patients is unclear, although likely to be predominantly White so included in sensitivity analysis of White ethnic group. §: Paper does not mention genotype quality control procedures, so reliability uncertain.†: Paper does not mention tests for population stratification, which is of concern since more than one ethnic group included.‡: Paper does not mention assessing compliance with treatment. ¥: Studies reported results assuming a dominant mode of inheritance. The effect size estimated is therefore for heterozygotes and mutant-type homozygotes combined versus wild-type homozygotes.

55 studies investigated association between CYP2C9*3 and stable dose. 30 were excluded: 27 [41], [124], [105], [42], [31], [66], [75], [17], [93], [95] [27], [21], [28], [44], [46], [47], [49], [58], [62], [81], [82], [85], [86], [104], [120], [123], [124] were also excluded from the meta-analyses for CYP2C9*2, for the same reasons, two [56], [113] since they reported medians rather than means, whilst one [19] presented data graphically only. Again, results from these studies are qualitatively consistent with the conclusions of the meta-analyses. Data for the remaining 25 studies are presented in Figure 3.

The pooled effect estimate for heterozygotes versus wild-types were similar for the Chinese and Japanese ethnic groups, and were statistically significant (−1.13 (−1.52;−0.75)mg/day and −1.18(−1.78;−0.58) mg/day respectively). For the two cohorts including Indian, Chinese and Malay patients, the pooled estimate was again significant but slightly greater (−1.47 (−2.16; −0.78) mg/day). For the White ethnic group, statistically significant pooled effect estimates were obtained for both genotype contrasts (−1.79(−3.30;−0.27) mg/day and −2.29(−2.98,−1.60)mg/day), although for the mutants versus wild-types contrast there was significant heterogeneity (I^2^∶75%).

It was suspected that studies investigating only one of CYP2C9*2 or *3 could be at risk of selective reporting on the basis that the hypothesis for genotyping both variants is the same. However, in the two studies reporting results for CYP2C9*2 but not *3 there were no patients with a *3 allele in one study [30] whilst for another [114] the one patient with the *1/*3 genotype and another with the *3/*3 genotype were excluded from analysis. Of the twelve studies reporting results for CYP2C9*3 but not *2, no *2 allele was present in any patients for seven [19], [53], [65], [102], [113], [119], [127], whilst a further four studies [50], [56], [61], [128] did not genotype for the *2 allele, which is not surprising since all eleven studies included patients of East Asian origin, within which the *2 allele has not been observed. In the twelfth study [25], it is unclear whether the *3 allele was also genotyped, therefore there is a risk of selective reporting in this study.

#### CYP2C9*2 and CYP2C9*3 combined and stable dose (0 copies vs. 1 copy vs. 2 copies of mutant allele)

**Figure 4:**
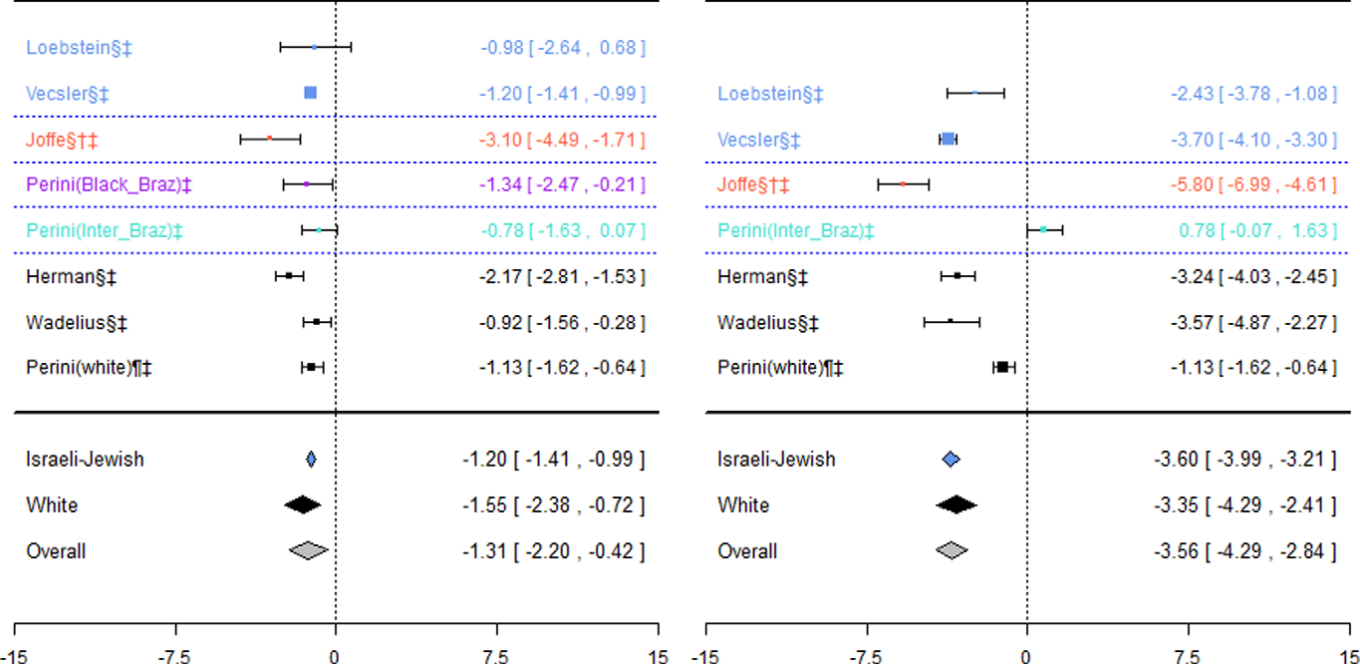
Forest plots for association between CYP2C9*2 and *3 combined and stable dose. Effect estimates are differences in means and 95% confidence intervals. ¶: Ethnicity of patients is unclear, although likely to be predominantly White so included in sensitivity analysis of White ethnic group. §: Paper does not mention genotype quality control procedures, so reliability uncertain.†: Paper does not mention tests for population stratification, which is of concern since more than one ethnic group included.‡: Paper does not mention assessing compliance with treatment.

Data for this association was available in six studies [39], [54], [89], [107], [112], [117] (Figure 4). Genotypes in each of these studies were in HWE.

For the Israeli-Jewish ethnic group, the pooled effect estimates for both genotype contrasts were statistically significant (−1.20(−1.41;−0.99) mg/day and −3.60(−3.99;−3.21) mg/day), as were those for the White ethnic group (−1.55(−2.38;−0.72) mg/day and −3.35(−4.29;-2.41) mg/day respectively).

#### CYP2C9*2 and CYP2C9*3 combined and stable dose (0 copies versus 1 or more copies of mutant allele)

Four studies [48], [68], [100], [103] investigated this association. Three were excluded: one [103] since it was a case-control study, with cases defined as those with high INR and controls as those with INR within the normal range, and therefore did not represent the general warfarin patient population, and two [68], [100] because insufficient data were reported. Their results were qualitatively consistent with the conclusions of the meta-analyses above. For the fourth study [48] there was a significant difference between the two groups, with those with one or more copies of a mutant-type allele requiring 1.11 (0.11, 2.09) mg less than those with no copies.

#### VKORC1 rs9934438 and stable dose

**Figure 5:**
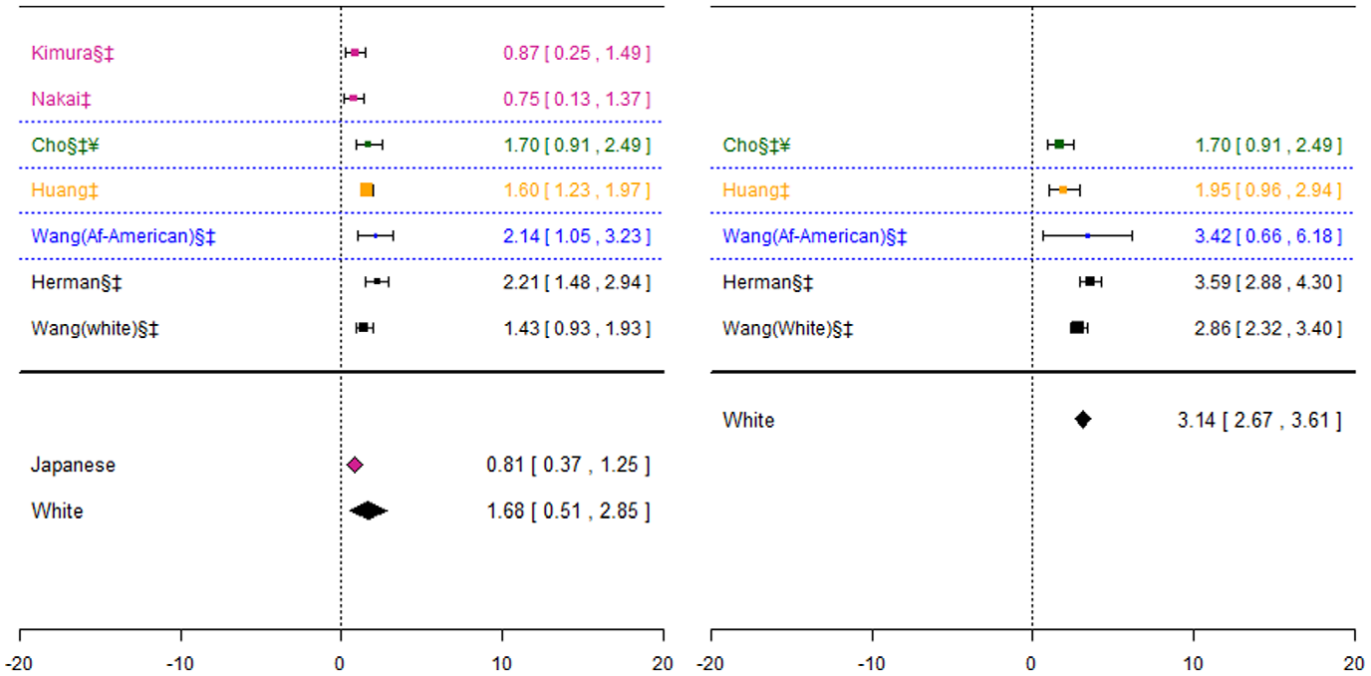
Forest plots for association between VKORC1 rs9934438 and stable dose. Effect estimates are differences in means and 95% confidence intervals. ¶: Ethnicity of patients is unclear, although likely to be predominantly White so included in sensitivity analysis of White ethnic group. §: Paper does not mention genotype quality control procedures, so reliability uncertain.†: Paper does not mention tests for population stratification, which is of concern since more than one ethnic group included.‡: Paper does not mention assessing compliance with treatment. ¥: Studies reported results assuming a dominant mode of inheritance. The effect size estimated is therefore for heterozygotes and mutant-type homozygotes combined versus wild-type homozygotes.

Fifteen studies investigated this association. Nine were excluded: five [20], [44], [83], [100], [106] reported insufficient data, two [36], [56] reported medians rather than means, one [105] presented data as the least square mean dose for each genotype category, after adjusting for clinical covariates, and one reported data graphically only [82]. Results from all these studies were qualitatively consistent with the conclusions of the meta-analysis. Data for the remaining six studies are summarised in Figure 5.

For the Japanese ethnic group, there was a significant difference in dose requirements between heterozygotes and wild-types, with wild-types requiring approximately 0.81(0.37, 1.25) mg/day more. There was also a statistically significant difference for the White ethnic group, with heterozygotes requiring 1.68(0.51, 2.85) mg/day more and mutant-types requiring 3.14(2.67, 3.61)mg/day more.

#### VKORC1 rs9923231 and stable dose

**Figure 6:**
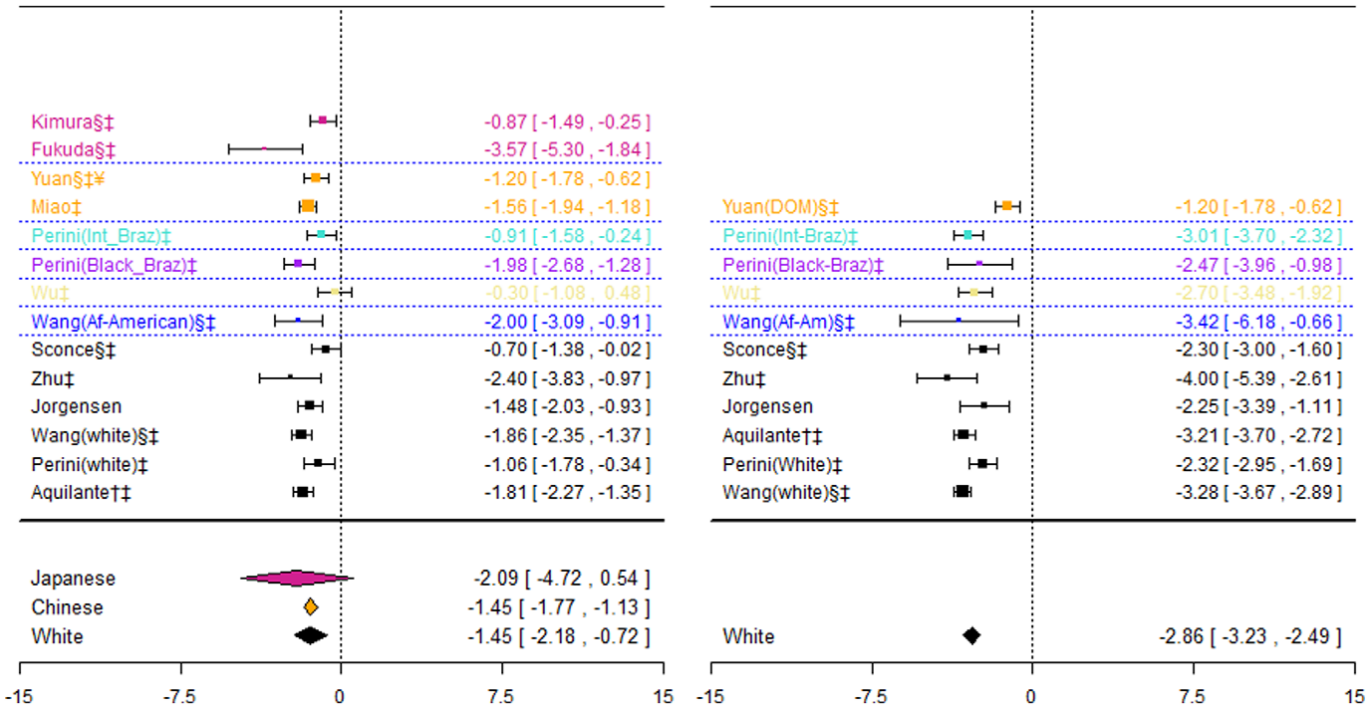
Forest plots for association between VKORC1 rs9923231 and stable dose. Effect estimates are differences in means and 95% confidence intervals. ¶: Ethnicity of patients is unclear, although likely to be predominantly White so included in sensitivity analysis of White ethnic group. §: Paper does not mention genotype quality control procedures, so reliability uncertain.†: Paper does not mention tests for population stratification, which is of concern since more than one ethnic group included.‡: Paper does not mention assessing compliance with treatment. ¥: Studies reported results assuming a dominant mode of inheritance. The effect size estimated is therefore for heterozygotes and mutant-type homozygotes combined versus wild-type homozygotes.

Twelve studies investigated this association. One [83] was excluded from meta-analyses as it reported insufficient data; however its results are consistent with those from included studies. Genotypes in all studies were in HWE, and data are summarised in Figure 6.

The pooled effect estimate for the white ethnic group was statistically significant for both genotype contrasts (−1.45(−2.18;−0.72) mg/day and (−2.86(−3.23;−2.49) mg/day respectively), although there was significant heterogeneity for both contrasts (I^2^∶64% and 60% respectively). The pooled effect estimate for the Japanese ethnic group was non-significant for heterozygotes versus wild-types, but heterogeneity was again substantial (I^2^∶88%), and there were no mutant type homozygotes present. For the Chinese ethnic group, the difference between wild-types and mutant-allele carriers combined was significant, with mutant-allele carriers requiring 1.45(1.12, 1.77) mg/day less.

#### VKORC1 rs7196161 and stable dose

One paper comprising two separate cohorts [101], including patients of Indian, Chinese and Malay ethnic background investigated this association. Combining data from both cohorts, the difference in stable dose was significant for both genotype contrasts (1.24(0.83; 1.65) mg/day and 2.79(1.93, 3.65) mg/day respectively). A further study also investigated this outcome, but was excluded as it reported insufficient data.

#### VKORC1 rs7294 and stable dose

**Figure 7:**
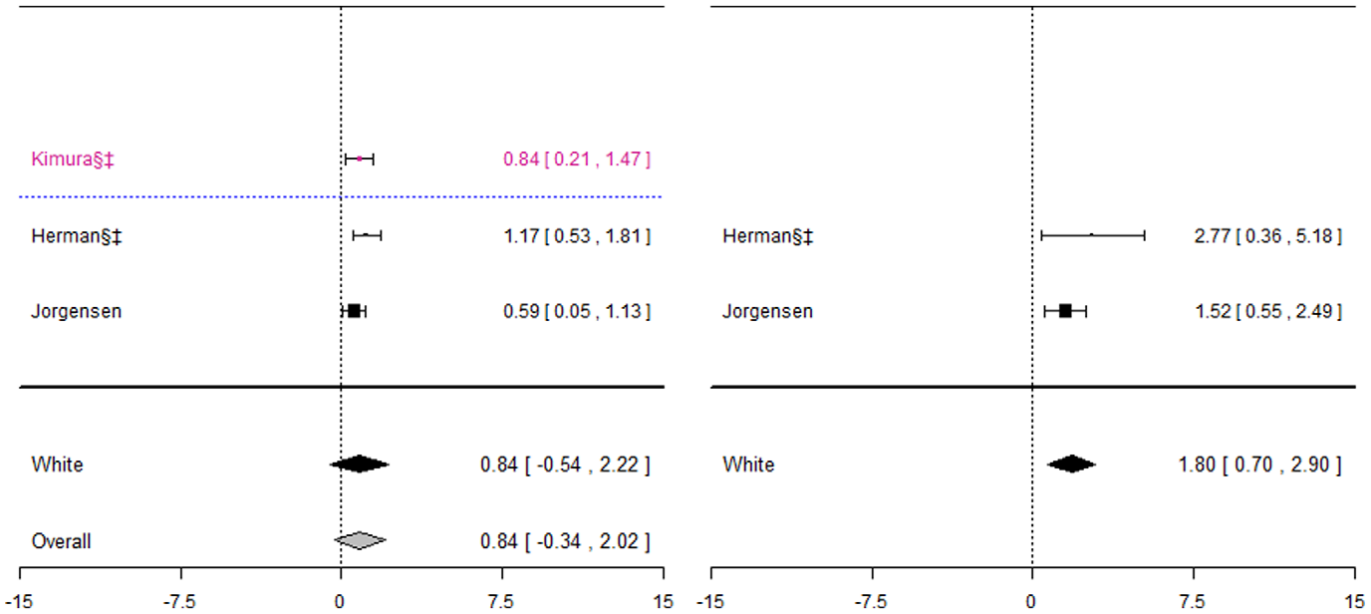
Forest plots for association between rs7294 and stable dose. Effect estimates are differences in means and 95% confidence intervals. ¶: Ethnicity of patients is unclear, although likely to be predominantly White so included in sensitivity analysis of White ethnic group. §: Paper does not mention genotype quality control procedures, so reliability uncertain.†: Paper does not mention tests for population stratification, which is of concern since more than one ethnic group included.‡: Paper does not mention assessing compliance with treatment.

Five studies [63], [82], [105], [125], [128] investigated this association. Two were excluded: one [105] reported the least square mean dose for each genotype category after adjusting for clinical covariates, one [82] presented data graphically only. Data for the remaining three studies are presented in Figure 7. For the White ethnic group, the pooled effect estimate was non-significant for heterozygotes versus wild-types, but was statistically significant for mutant-types versus wild-types (1.80(0.70; 2.90) mg/day).

#### VKORC1 rs8050894 and stable dose

Five studies investigated the association with rs8050894, however three were excluded: one [105] reported the least square mean dose for each genotype category after adjusting for clinical covariates, one presented data graphically only [82], and one reported insufficient data [83]. Of the remaining two studies, patients in one [51] were a subset of African-American patients from the other [88]. In the larger of these two, the difference was not statistically significant for either genotype contrast. This study also included a group of White patients, and the difference was significant for both genotype contrasts, with heterozygotes requiring 1.86(1.34, 2.38) mg/day less than wild-types and mutant-types requiring 3.14(2.72, 3.36) mg/day less.

#### Other VKORC1 SNPs investigated in more than one study for association with stable dose

A further five studies [41], [63], [82], [83], [105] investigated association with rs2359612. However, one [105] reported the least square mean dose for each genotype category after adjusting for clinical covariates rather than the mean stable dose whilst another two [41], [82] provided data graphically only. A further study [83] reported insufficient data to be considered for meta-analysis.

In addition, one study conducted a GWAS analysis and therefore investigated several SNPs within the VKORC1 gene. However, as the study report only presented p-values for the most statistically significant SNPs there was insufficient data to include this study in the meta-analyses.

#### CYP2C9*2 and *3 combined and time to stable dose (no mutant-type alleles versus at least one)

Nine studies [17], [32], [48], [49], [61], [63], [79], [82], [118] investigated this outcome. Five were excluded: one [79] presented data graphically only, another two [48], [61] presented median or mean time to event rather than hazard ratios, one [49] presented hazard ratios from an adjusted analysis only, two studies [82], [118] classed a mutant allele as any amongst several SNPs, including *2 and *3. Genotypes for the remaining four studies were in HWE, but the pooled hazard ratio was not significant for either of the two included ethnic groups, White and African-American (forest plot not shown).

#### VKORC1 rs9934438 and time to stable dose

Three studies investigated this outcome, however one [61] was excluded as only median time to stable dose was reported as opposed to hazard ratios. Of the remaining two studies, one included patients of African-American and of European-American ethnic background [82], whilst the other [118] included patients of Korean ethnic background. The hazard ratios were not statistically significant in any of the ethnic groups.

#### CYP2C9*2 and bleeding events

**Figure 8:**
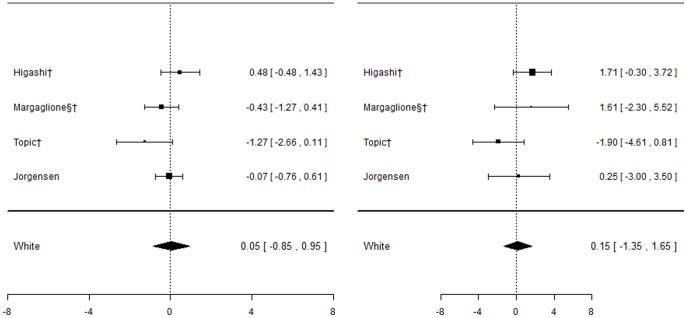
Forest plots for association between CYP2C9*2 and bleeding events. Effect estimates are odds ratios and 95% confidence intervals. ¶: Ethnicity of patients is unclear, although likely to be predominantly White so included in sensitivity analysis of White ethnic group. §: Paper does not mention genotype quality control procedures, so reliability uncertain.†: Paper does not mention tests for population stratification, which is of concern since more than one ethnic group included.‡: Paper does not mention assessing compliance with treatment.

Five studies [32], [43], [63], [116], [120], all including patients of White ethnic origin, investigated this association (Figure 8). Genotypes were in HWE for all studies.

Even though some of the studies undertook two or more separate analyses, each for a different severity of bleed (Table 5), all bleeding events were combined into a single analysis for meta-analysis. A sensitivity analysis was also undertaken excluding the study by Higashi et al. [32] that only counted serious or life-threatening bleeds as a bleeding event. Two of the studies [43], [120] included exactly the same patients, and so data from the first only was included. The pooled effect estimate was not significant for either of the two genotype contrasts; however heterogeneity was significant for the mutant versus wild-types contrast (I^2^∶59%).

#### CYP2C9*3 and bleeding events

**Figure 9:**
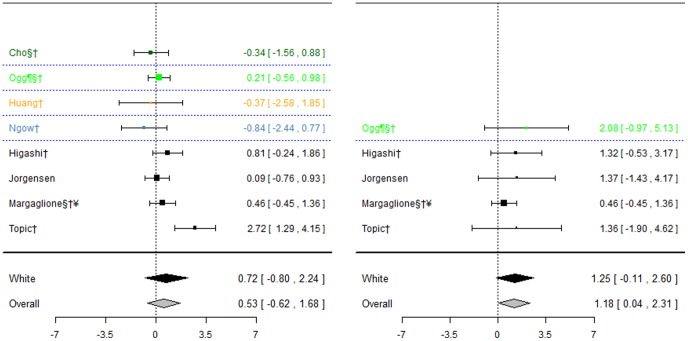
Forest plots for association between CYP2C9*3 and bleeding events. Effect estimates are odds ratios and 95% confidence intervals. ¶: Ethnicity of patients is unclear, although likely to be predominantly White so included in sensitivity analysis of White ethnic group. §: Paper does not mention genotype quality control procedures, so reliability uncertain.†: Paper does not mention tests for population stratification, which is of concern since more than one ethnic group included.‡: Paper does not mention assessing compliance with treatment.

Eight studies investigated this association (Figure 9), five of which [32], [43], [63], [116], [120] also investigated CYP2C9*2. Again, for the White ethnic group, the pooled effect estimate was not significant for either of the genotype contrasts, although when including the one study where ethnicity was unclear in a sensitivity analysis, a significant effect was observed for mutant versus wild-types (odds ratio: 1.18(0.04; 2.31)) only.

#### CYP2C9*2 and *3 combined and bleeding events

Seven studies [40], [48], [49], [79], [82], [87], [117] investigated association between CYP2C9*2 and *3 combined and bleeding events. Two were excluded: one [49] reported the hazard ratio for bleeding risk as opposed to the number of bleeding events, and one [82] classed a mutant allele as a mutant allele at any of several SNPs, including *2 and *3. In a pooled analysis of the two studies including White patients [79], [117], there was no significant difference between heterozygotes and wild-types. The remaining three studies all included patients of a different ethnic background. Only three studies included mutant-type patients, and they all included patients from different ethnic backgrounds.

**Figure 10:**
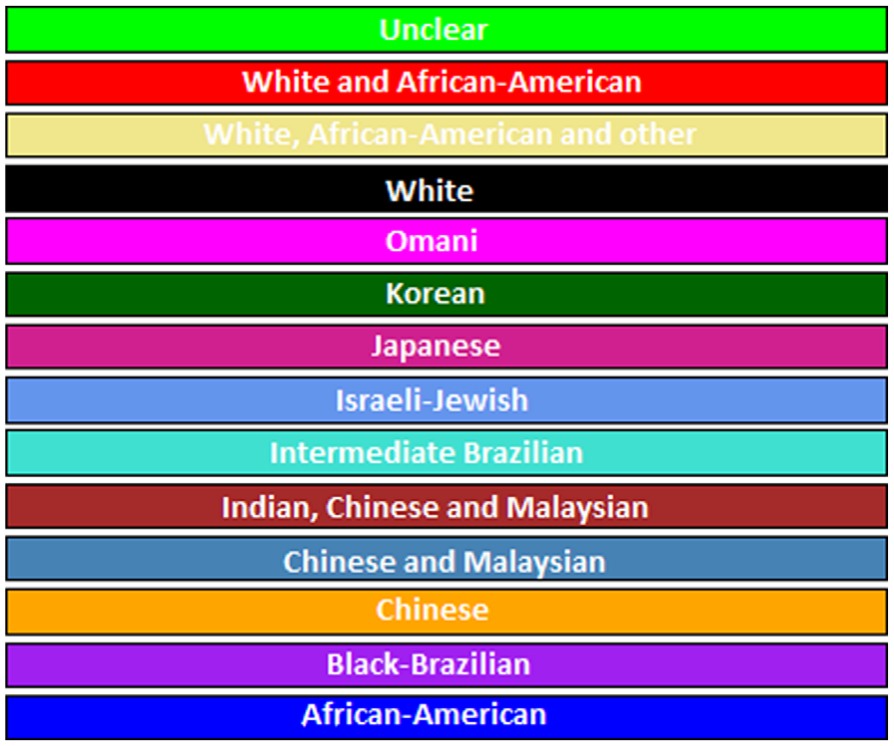
Key to colours used in forest plots.

#### VKORC1 rs9934438 and bleeding events

Two studies investigated this outcome, one [61] conducted in a Chinese population and the other [84] in a white and African-American mixed population. The odds ratio for the heterozygotes versus wild-types was not significant in either study, whilst there were no events in the mutant-type patient groups.

#### CYP2C9*2 and *3 combined and INR>4 during the first week

Two studies [63], [95] investigated association between variants in CYP2C9 and the occurrence of INR>4 during the first week of treatment. One included patients of White ethnic background whilst the ethnicity of patients in the other study was unclear. Effect estimates were not significant in either study.

#### CYP2C9*2 and *3 combined and time to therapeutic INR (no copies versus 1 or more copies of mutant-type allele)

Seven studies [32], [40], [63], [79], [82], [95], [120] investigated this association. Four were excluded: one [79] presented data graphically only, another two [95], [120] presented median time to therapeutic INR rather than hazard ratios, and one [82] classed a mutant allele as a mutant allele at any of several SNPs, including *2 and *3. Of the three remaining studies, two included patients of White ethnic origin and the third included patients of White, African-American and other unknown ethnic background. Genotypes in all studies were in HWE, and the effect estimate was not significant for either of the two ethnic groups. A further study including White, African-American and Hispanic patients [87] also investigated this association, but tested the two genotype contrasts separately, although the hazard ratios for both were again not significant.

#### CYP2C9*2 and *3 combined and time in therapeutic INR range

Four studies investigated this association [48], [49], [79], [96]. Two provided data graphically only [49], [79], and one provided insufficient information to include it within a meta-analysis [96].

## Discussion

The evidence base for the effect of CYP2C9 and VKORC1 genotype on response to warfarin is substantial; however navigating through the literature to ascertain what effect a particular variant has on which outcomes in which patients, and to what extent, is difficult. This is not least due to significant variability between studies in terms of ethnic background of participants, study design, statistical analysis approaches, methodological rigour, and choice of outcomes and their definition. To address this, a systematic review was undertaken which provided a structured framework within which all evidence accumulated to date could be identified and methodically allocated to a particular patient subgroup. A key element was a rigorous assessment of methodological quality, in accordance with a previously published checklist [11]. This enabled each piece of evidence to be considered in light of the robustness of the study from which it was derived, with particular caution taken in the event that the assessment suggested a significant risk of bias.

### Methodological Quality and Risks of Bias

As anticipated, the methodological rigor of studies was highly variable, with many areas of concern. Most studies were significantly smaller than typically required to provide sufficient power, and the reader was left uninformed about the likelihood of false-negatives in all studies due to the lack of reporting of a priori power calculations. There was also uncertainty around the reliability of genotypes in several studies, since 68% did not describe any genotype quality control procedures. Further, there was a risk of bias from non-random missing genotype data, which is highly probable since heterozygotes are notoriously more difficult to call than homozygotes, due to a lack of information on missing data. Further many of the studies were at risk of confounding from population differences with only 57% of the 37 studies including more than one ethnic group adjusting for this in their analyses.

Importantly, our review identified a significant risk of selective reporting amongst pharmacogenetic studies. This risk comes from several different sources including the huge number of known genetic variants available to investigate, the several possible assumptions regarding the underlying mode of inheritance available to those analysing the data and since the choice of outcomes and definitions in pharmacogenetic studies is often subjective. The large variability in outcome definitions also caused difficulties in replicating findings, comparing results between studies and also introduced heterogeneity to the meta-analyses. This was a particular issue for the outcome of stable dose where 34 different definitions were used across 55 studies, and a further 21 studies failed to provide any definition and made interpreting the results of the meta-analyses difficult. Ioannidis et al. [129] made similar findings when examining the variability of definitions of outcomes across studies addressing the association of the Arg16Gly and/or Gln27Glu polymorphisms of the β2-adrenergic receptor gene with clinical response to β2-agonist therapy in asthma, suggesting that this may be a widespread problem across the field of pharmacogenetics.

Our assessment of the methodological rigour of included studies was intentionally qualitative, since quantitative methods which typically weigh each issue of quality equally was not deemed appropriate on the basis that a study weak in terms of one very important issue of quality could score better than a study found to be weak in terms of several, more trivial, issues [130]. However, one consequence of this is that the quality of a study is not readily recognisable from a single summary score, and studies cannot be ordered in terms of overall rigour. It is also important to note that we were only able to assess the quality of studies based on information published in the study reports, and this will always be a limitation for investigators involved in systematic reviews.

### Meta-analyses

Where possible, meta-analyses were undertaken in an attempt to improve power to estimate a genetic effect. This also provided an opportunity for potential sources of heterogeneity to be investigated. The advanced meta-analysis methods adopted allowed more precise estimates of effect than undertaking two separate meta-analyses (one for heterozygotes versus wild-type and one for mutant-type versus wild-type) since they accounted for the inherent correlation between the two genotype contrasts, whilst not requiring a specific assumption regarding mode of inheritance. They also enabled studies making different assumptions regarding the underlying mode of inheritance to be included in the same meta-analysis, thus improved power.

In terms of the conclusions arrived at in the meta-analyses, no significant associations were found between CYP2C9*2 and stable dose requirements for either Asian or white patients. However, for the group including white and African-American patients, the difference between heterozygotes and wild-types was statistically significant with heterozygotes requiring almost 2 mg/day less. Significant associations were observed between CYP2C9*3 and stable dose for the White, Chinese, Japanese and a mixed Indian, Chinese and Malaysian population. For the heterozygotes versus wild-type contrast the largest difference was observed for the white population with heterozygotes requiring almost 1.80 mg/day less. For the Japanese and Chinese populations the estimates were similar at around 1.20 mg/day less, with the difference for the mixed Indian, Chinese and Malaysian population being in between these two estimates at around 1.50 mg/day less. An effect size for the other genotype contrast was only estimable in the white population, with mutant types requiring almost 2.30 mg/day less.

Holding two copies of the mutant allele at the CYP2C9*3 SNP was also found to increase the risk of bleeding, with the odds ratio for mutants relative to wild-types estimated at 1.18. However, the effect was only significant when combining data across all ethnic groups. Bleeding events are relatively rare, and therefore failure to detect an association in the stratified analyses or for the other genotype contrasts investigated may be as a result of insufficient power and the presence of true associations should not be discounted.

Some studies did not differentiate between the CYP2C9*2 and *3 SNPs, comparing those with no copies of either *2 or *3 mutant-type alleles to those with one and two copies respectively. Again, significant associations were observed for both the white and Israeli-Jewish ethnic groups. For the former, the difference between heterozygotes and wild-types was just over 1.50 mg/day less whilst the difference between mutant-types and wild-types was much larger at 3.35 mg/day less. For the latter, a slightly smaller difference was observed for the first genotype contrast at 1.20 mg/day less although the difference for the second genotype contrast was slightly larger at 3.60 mg/day less. Similar estimates were observed when combining all studies in a single meta-analysis.

A significant association was also observed between the VKORC1 rs9923231 SNP and stable dose in both the white and Chinese ethnic groups, with heterozygotes requiring 1.45 mg/day less than wild-types in both groups. It was only possible to calculate a pooled estimate for the difference between mutant-types and wild-types in the white ethnic group, and this was found to be almost double this at just under 2.90 mg/day less.

Further, a significant difference was also observed between mutant and wild-type homozygotes at the rs7294 SNP in the white population, with the former requiring 1.80 mg/day more than the latter. A similar estimate was obtained when including data from all studies in a single meta-analysis.

Often, heterogeneity was significant for one of the genotype contrasts but not the other. This is surprising, as it is expected that sources of heterogeneity would influence both contrasts to the same extent. One possible explanation could be the small number of patients in some genotype groups (particularly the mutant type homozygote group) of some studies. We acknowledge that our method of exploring potential reasons for heterogeneity using sensitivity analyses is rather simplistic, and a more formal exploration of sources of heterogeneity could be achieved by meta-regression. However, since the number of studies in each meta-analysis was small this approach was not considered here.

Further, it is worth noting that only aggregate meta-analyses are considered here, however an alternative approach would be to conduct an individual patient data (IPD) meta-analysis where raw data collected within each study is obtained and analysed using methods such as multi-level modelling to account for study-level effects. Such methods would allow outcomes to be standardised across datasets and would also facilitate adjustment for between-study heterogeneity since patient-level as well as study-level variability could be accounted for. It would also overcome, at least in part, the issue of bias from the selective reporting of both outcomes and genetic variants. Since conducting an IPD meta-analysis is inherently resource-intensive we were unable to consider this approach here, however given the additional benefits it can offer it may be worth considering in the future for warfarin and other areas of pharmacogenetic research. The work of the International Warfarin Pharmacogenetic Consortium on developing a dose prediction model [131] is an example of successfully utilising IPD, although data from only a subset of all conducted warfarin pharmacogenetic studies contributed to this analysis.

The advanced meta-analysis methods applied may improve power and precision, however they do rely on particular pieces of summary data being reported in the study publication. This data includes the numbers of patients and events in each of the three genotype groups for a binary outcome and numbers of patients, and means and standard deviations per genotype group for a continuous outcome. Some of this data was omitted from the report of some studies, whilst others provided only p-values for the associations investigated, or merely stated that they were non-significant. Unfortunately, all these studies had to be excluded from the meta-analyses, although where it was possible to assess so the results were qualitatively consistent with included studies. It was also necessary to exclude some studies due to uncertainty about outcome definition and the ethnic origin of participants.

### Recommendations for those Conducting Pharmacogenetic Research

Given the sparse reporting and concerns regarding methodological quality observed in some studies, we recommend that priority should be given towards improving the reporting and methodological quality of pharmacogenetic studies, since even the most sophisticated methods of analysis will not compensate for lack of data and poor methodology. In this regard, we would make a number of recommendations regarding the conduct and reporting of pharmacogenetic studies, with a view to making such studies more amenable to systematic reviews and meta-analyses in the future. Otherwise, the literature will provide an incomplete picture of the accumulated evidence on the associations of interest and, as such, meta-analyses may be biased. These recommendations are as follows:

- Studies should adhere to rigorous methodological quality. Guidance in this regard is given in Jorgensen and Williamson [11];
- So that any quality assessment based on the published paper is a fair reflection of the study’s true underlying methodological quality, researchers are encouraged to be as transparent as possible in their study reports in terms of what has been done;,
- The number of patients in each genotype group should be reported;
- For binary outcomes, the number of events in each genotype group should be reported;
- For continuous outcomes, the means and standard deviations should be reported for each genotype group separately;
- The ethnicity of included patients should be reported;
- In the event that a study includes more than one ethnic group, the summary data specified in i)–iii) above should be provided per ethnic group;
- To minimise the risk of selective reporting, researchers should ensure complete transparency in terms of how their study is conducted by publication of protocols in advance and full reporting of all variants and outcomes investigated and of all analysis approaches undertaken in the study report.
- Consensus should be reached between experts in the fields on a core set of outcomes that should be investigated in any pharmacogenetic study of a particular treatment, together with definitions. An effective way of achieving this is to encourage communication between groups investigating the same association such that a prospective meta-analysis can be planned, with outcomes and methods synchronised between the research groups. This would facilitate meta-analyses by reducing heterogeneity, increase the number of studies combined in a single meta-analysis, as well as minimise the risk of selective reporting of outcomes. It would also facilitate the work of consortia such as the International Warfarin Pharmacogenetic Consortium [131], where several international datasets contribute to a single, large, association study.

These recommendations are primarily aimed at improving reporting of pharmacogenetic studies, specifically with a view to facilitating future systematic reviews and meta-analyses of pharmacogenetic studies, however we also recommend that the reporting guidelines ‘STREGA’ [132], developed primarily with gene-disease association studies in mind, are also referred to. It is appreciated, with researchers increasingly using a genome-wide approach to their investigations thus collecting data on a huge number of SNPs in any given study, that the level of detail recommended in ii)–vi) above can be problematic due to limited journal space, however this reporting could be facilitated by the use of supplementary data, accessible electronically.

## Methods

A protocol describing methods for the review was published on the HuGENet database [133] in advance.

### Inclusion Criteria

Participants were already established on or commencing warfarin treatment and genotyped for CYP2C9 or VKORC1 variants to investigate their effect on treatment response. Prospective and retrospective cohort studies, case control studies and randomized controlled trials were included. Case studies were excluded. Only studies published as journal articles in the English language were included.

### Outcomes

The three co-primary outcomes were stable maintenance dose, time to achieving stable maintenance dose and bleeding events. Secondary outcomes were INR greater than four during the first week, time to achieving therapeutic INR, proportion time spent within therapeutic range, warfarin sensitivity (1.5 mg or less on three successive clinic visits), and warfarin resistance (10 mg or more on three successive clinic visits).

### Search Strategy

MEDLINE was searched on 30 September 2009 applying the search strategy summarised in Table 1. Reference lists of all identified studies were scrutinized for further papers of potential interest. A list of titles and abstracts for identified studies were reviewed with any obviously irrelevant studies removed. For the remaining papers, full text articles were retrieved and each assessed individually for eligibility. This process was undertaken by two reviewers (ALJ and RF/JO) independently with differences resolved by discussion.

### Data Extraction

Data were extracted in accordance with the methods set out in the Cochrane Handbook [134], onto data extraction forms which were piloted on the first five studies. This included information pertinent to assessing that review inclusion criteria had been met, patient demographics, outcome data, study design and data for assessing methodological quality. Assessment of methodological quality was qualitative, and undertaken in accordance with Jorgensen and Williamson [11]. Papers were randomly allocated between two reviewers for data extraction, although some initial training was undertaken to ensure consistency.

### Statistical Analysis

For each SNP-outcome combination investigated by more than one study, a meta-analysis was undertaken. Forest plots were prepared, stratified by ethnicity as recommended by HuGENet [130], for each genotype contrast separately (heterozygotes versus wild-type homozygotes (‘wild-types’) and mutant-type homozygotes (‘mutant-types’) versus wild-types). A key to colours used in the forest plots is provided in Figure 10. For continuous outcomes, the difference in means was estimated between two genotype groups; for binary outcomes the odds ratio was estimated.

To estimate a single pooled effect for each genotype contrast, the genetic model-free approach of Minelli et al. [12] was applied to each ethnic group separately, and random effects assumed. This method models the two effect estimates from each study as being bi-variate normally distributed, thus allowing the two effect sizes to be estimated separately whilst still accounting for the inherent correlation between them. It does not require a specific assumption to be made in advance regarding the underlying mode of inheritance, but rather estimates this from the data. Further, this mode is not restricted to one of the classic modes of dominant, additive or recessive.

The method was applied in Stata (v9.2) and relied on each study contributing data on both genotype contrasts. Where no mutant-types were present in a study and the outcome was continuous, the mean outcome for that genotype group was estimated as the mean for the mutant-type group across all other studies within the same ethnic group, whilst the standard deviation was set to be very large, ensuring the study contributed almost nothing to the analysis. Where the outcome was binary, this problem was overcome by adding 0.5 to each cell of the hypothetical contingency table. Minelli et al. acknowledge that the between-study covariance is poorly estimated in their proposed method when the number of studies is small [12], and therefore as they recommend sensitivity analyses were conducted assuming various fixed values for the between-study correlation. Unless otherwise stated, results were robust to this variation. Where mutant-types were not present in any study within an ethnic group, or where all studies in an ethnic group assumed a dominant mode of inheritance, a standard random-effects approach [135] was used instead. Prior to applying the method of Minelli et al. the data were explored graphically to confirm that the necessary assumption of a constant mode of inheritance across all studies was reasonable. If not, a joint pairwise bi-variate approach was employed instead [12].

Where an ethnic group included studies that differed in terms of their assumption about mode of inheritance (e.g. where some made no assumption whilst others assumed a dominant mode of inheritance), the method of Salanti et al. [13] was used instead to obtain the pooled effect estimates. This utilises the genetic model-free approach of Minelli et al. whilst allowing studies making different assumptions regarding the underlying mode of inheritance to be included together in a single analysis, thus maximizing power. This method was applied in WinBUGS [136] using a chain length of 100,000 after discarding the first 10,000 to allow for convergence. Each analysis was repeated three times using different initial values, and compared to check for convergence. Where studies assumed a dominant mode of inheritance in their analysis, the effect estimate for heterozygotes and mutant homozygotes combined versus wild-type homozygotes has been included on the forest plots for both genotype contrasts i.e. for these studies, the effect estimate will appear the same on both forest plots.

To assess for heterogeneity, the I^2^ statistic was calculated and forest plots inspected. Where heterogeneity was significant (I^2^>50%), differences in methodological quality was considered as a potential contributing factor. To investigate this, sensitivity analyses were conducted excluding studies with questionable methodological rigor, with reference to two particular issues of concern: failure to report usage of any genotype quality control procedures, and failure to report testing for the presence of population stratification.

A test for Hardy-Weinberg Equilibrium (HWE) was undertaken within each study separately. Where genotypes deviated from HWE (p<0.001) a sensitivity analysis was conducted excluding that study.

Unless otherwise stated, conclusions from all sensitivity analyses were consistent with the main analyses.

Studies were investigated for evidence of overlapping datasets by sorting them according to geographic region, then date, and scrutinising author names and affiliations. Any two studies found similar with regard to any of these were scrutinised to identify whether the same patients had been included in both, in which case only the largest study was included in the meta-analysis.
